# World’s first surgical repair of bilateral common iliac artery agenesis

**DOI:** 10.1308/rcsann.2025.0077

**Published:** 2026-01-12

**Authors:** T Ali, O Woolf, B Farquharson

**Affiliations:** Frimley Health NHS Foundation Trust, UK

**Keywords:** Iliac artery abnormalities, Aorto-bifemoral bypass, Vascular surgery, Collateral circulation, Surgical revascularisation, Embryology of iliac arteries

## Abstract

We present a rare case of a 23-year-old woman with congenitally absent bilateral common iliac arteries, identified incidentally during a computed tomography scan of the abdomen. Imaging revealed occlusion of the aorta below the renal arteries with flow to the lower limbs provided by hypertrophied collaterals. Despite initial conservative management, the patient experienced worsening claudication and functional limitations, prompting surgical revascularisation. An aorto-bifemoral bypass using an axillary–bifemoral graft was performed with successful recovery – the first documented case of surgical repair of this anatomical abnormality, and a novel use of the Gore heparin-bonded graft. Two years post-surgery, the patient was symptom-free. This case highlights the rarity of common iliac artery agenesis, the compensatory role of collateral circulation, and the potential for successful surgical intervention.

## Background

Anomalies of iliac artery development are extremely rare. In a review of 8,000 symptomatic patients undergoing angiography, only 6 cases of iliofemoral malformations were reported.^1^ We present a unique case of a 23-year-old woman with congenitally absent bilateral common iliac arteries, including her clinical presentation, investigations, surgical management and long-term outcome.

## Case report

A 23-year-old woman presented with a 12-month history of hypoglycaemia and dizziness of unknown cause. She underwent a computed tomography (CT) scan of the abdomen to rule out insulinoma, which showed an incidental finding of atresia in the lower aorta and iliac arteries.

When questioned, the patient reported 12 months of bilateral lower limb claudication, occurring after approximately 10 minutes of walking, or 2 minutes of walking uphill. On examination, a palpable aorta was noted above the umbilicus, with no discernible pulses below this point.

A CT angiogram of the aorta revealed occlusion of the aorta 2cm below the left renal artery (seen on the sagittal view; Figure 1). The common iliac arteries were absent, and both the internal and external iliac arteries were reconstituted bilaterally (seen on the reconstruction; Figure 2). Collateral blood flow to the lower limbs was supplied by hypertrophied inferior epigastric, internal mammary and ileo-lumbar arteries, as well as through the hemorrhoidal plexus.

**Figure 1:**
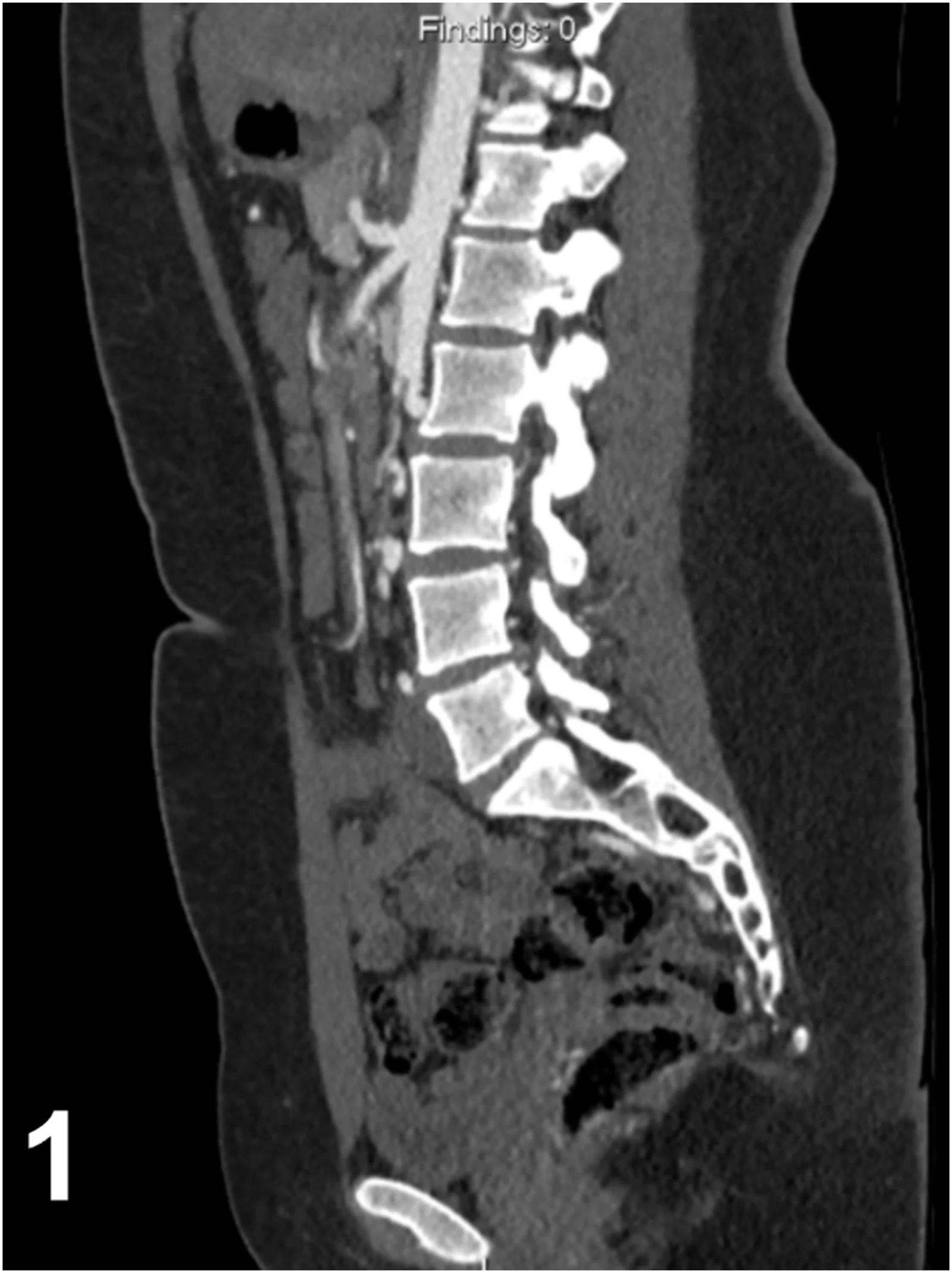
CT angiogram (sagittal view) showing the occlusion of the aorta 2cm below the left renal artery

**Figure 2:**
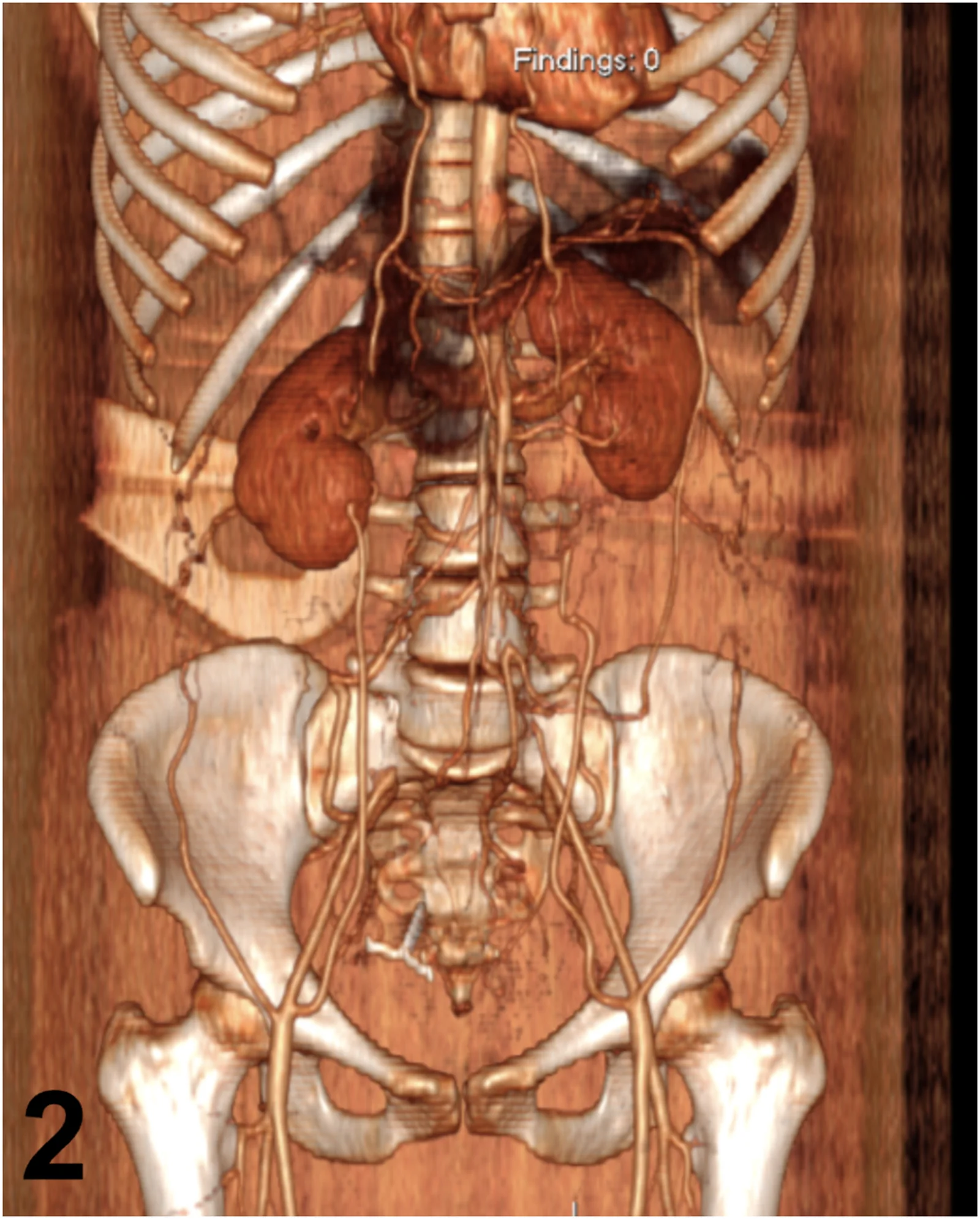
Reconstruction showing congenitally absent iliac arteries reconstituted bilaterally

Initial management involved lifestyle modifications, including cessation of smoking and weight loss. The patient was re-evaluated after 12 months. At this point, she described worsening claudication: 10 minutes on flat surfaces, 5 minutes uphill and only 1–2 minutes of running before experiencing pain. The patient reported that her symptoms were significantly life-limiting and expressed personal goals, including travelling and completing the Inca Trail. Considering the functional limitations imposed by her condition, and following discussion at a vascular multidisciplinary team meeting, a decision was made to proceed with surgical revascularisation.

Under general anaesthesia, the surgeon performed an aorto-bifemoral bypass. A midline laparotomy and two skin crease incisions in the groin were used to access the vascular structures. Upon opening the retroperitoneum, the aorta was found to have a small calibre of 9mm in diameter, terminating in an atretic section that gave rise to the median sacral artery. The left mesocolon showed a pulsatile mesenteric artery (Drummond’s artery). The renal arteries were carefully identified, and the renal vein was divided between Liga ties and oversewn with 4.0 Prolene sutures.

After identifying the common femoral arteries in the groin, a retroperitoneal tunnel was created for the placement of an 8mm trifurcated reinforced polytetrafluoroethylene graft. An axillary–bifemoral graft was utilised, which is unusual for aortic operations. The reason for using this type of graft was that the aorta was too small in calibre for a conventional aorto-bifemoral graft. The graft was anastomosed to the infrarenal aorta using 5/0 Prolene sutures, and to the common femoral arteries with 7/0 Prolene sutures. The surgical sites were closed in layers. The patient awoke from surgery without complications, and pulses were palpable in both feet. She was extubated successfully and transferred to the intensive care unit for 24h monitoring, requiring a lignocaine infusion for pain management.

Two weeks postoperatively, the patient was well, with palpable anterior tibial pulses and no further claudication. A repeat angiogram performed at three months showed that the graft remained patent (Figure 3), and the patient’s exercise tolerance had significantly improved, with no residual pain. Femoral and peripheral pulses were strong bilaterally.

**Figure 3:**
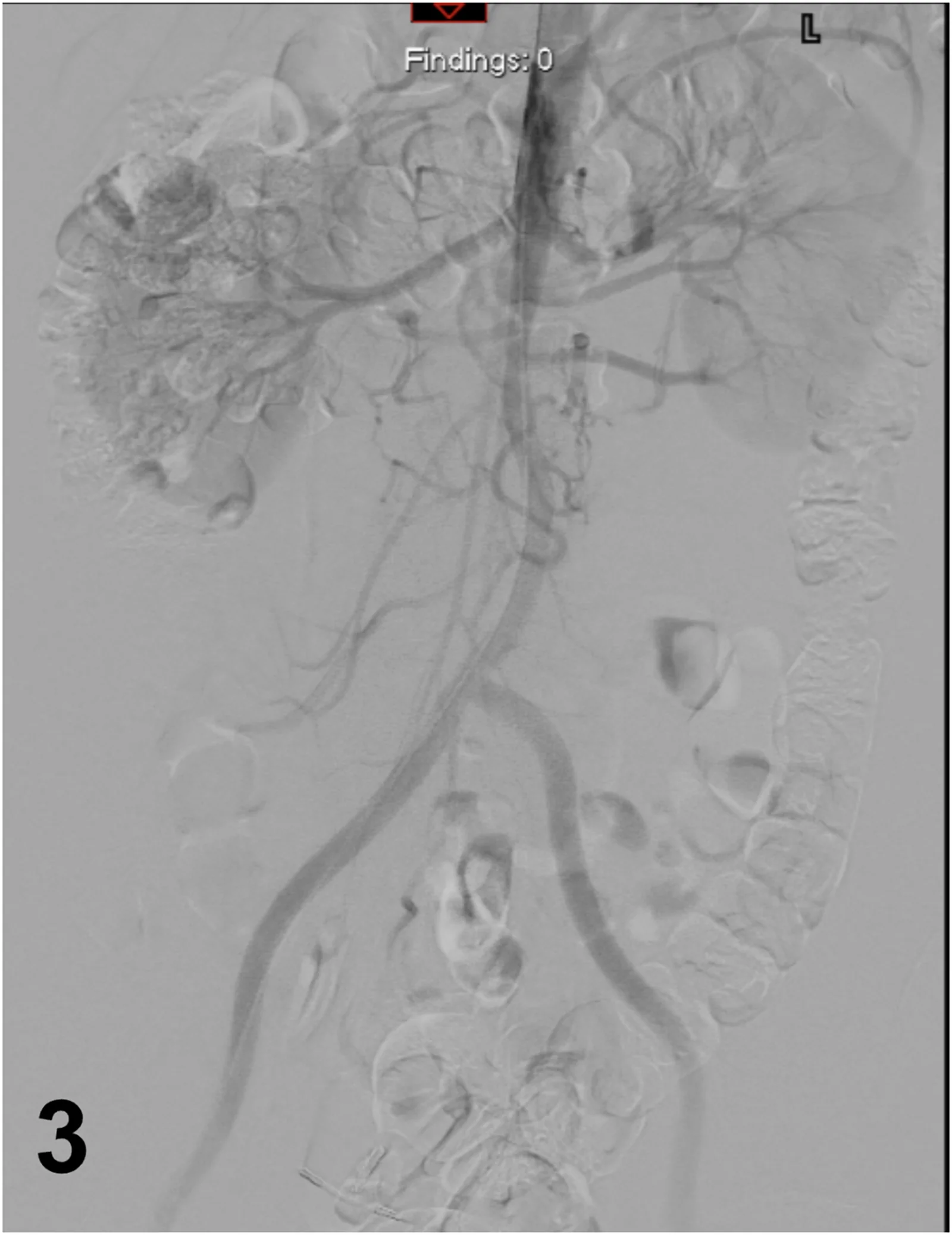
Repeat angiogram showing the patent graft

Two years post-surgery, the patient had made significant progress. She had lost considerable weight, secured new employment and was able to engage in running. Her claudication had completely resolved, and she was thriving.

## Discussion

Owing to the rarity of common iliac artery abnormalities, the medical literature primarily consists of isolated case reports. We found five reports of bilateral common iliac artery agenesis, all of which were incidental findings. In four instances, the aorta terminated into two external and two internal iliac arteries with no symptoms recorded.^2–5^ In the fifth case, the anatomy was similar to that of our patient, with absent common iliac arteries bilaterally and blood supply to the lower limbs provided by collateral arteries, which contributed to the reconstitution of flow into the external iliac arteries.^6^ All five cases were managed conservatively. To date, there have been no reported cases of surgical management of bilateral iliac artery atresia.

There are no documented risk factors or conditions associated with common iliac artery agenesis.

### Embryology

Infrarenal aortic atresia is an exceptionally rare congenital vascular anomaly characterised by the absence or occlusion of the abdominal aorta below the renal arteries. Embryologically, the descending aorta develops from the fusion of the paired dorsal aortae during early gestation. During weeks 4 to 6 of development, the paired dorsal aortae fuse to form the midline abdominal aorta, while the fifth lumbar intersegmental arteries evolve into the common iliac arteries.^7^

Disruptions in this process, such as incomplete fusion, abnormal regression or failure of recanalisation, can result in segmental atresia. Specifically, infrarenal aortic atresia may arise from aberrant development or regression of the dorsal aorta caudal to the renal arteries. Alternatively, intrauterine vascular insults, like thrombosis or hypoperfusion, could lead to secondary atresia of the infrarenal segment. Owing to the rarity of this condition, its precise embryological mechanisms are not completely understood.

In common iliac artery agenesis, there is a complete absence of the common iliac artery. It is typically due to aberrant embryologic development of the fifth lumbar intersegmental artery. It may be unilateral or bilateral and is often compensated by well-developed collateral circulation (as in our case) from the lumbar, mesenteric or hypogastric arteries, allowing for distal perfusion despite the absence of the primary vessel.^8^

## Conclusion

This case highlights an exceptionally rare vascular abnormality. Despite the presence of extensive collateral circulation, the patient experienced progressive functional limitation, ultimately requiring surgical revascularisation via aorto-bifemoral bypass, using a novel technique. This case supports a proactive approach to surgical intervention when symptomatic deterioration occurs.
